# Deriving the Hawking Temperature of (Massive) Global Monopole Spacetime via a Topological Formula

**DOI:** 10.3390/e24050634

**Published:** 2022-04-30

**Authors:** Junlan Xian, Jingyi Zhang

**Affiliations:** Center for Astrophysics, Guangzhou University, Guangzhou 510006, China; xianjlphys@yeah.net

**Keywords:** topology, Euler characteristic, Hawking temperature

## Abstract

In this work, we study the Hawking temperature of the global monopole spacetime (non-spherical symmetrical black hole) based on the topological method proposed by Robson, Villari, and Biancalana (RVB). By connecting the Hawking temperature with the topological properties of black holes, the Hawking temperature of the global monopole spacetime can be obtained by the RVB method. We also discuss the Hawking temperature in massive gravity, and find that the effect of the mass term cannot be ignored in the calculation of the Hawking temperature; the corrected Hawking temperature in massive gravity can be derived by adding an integral constant, which can be determined by the standard definition.

## 1. Introduction

The discovery of gravitational waves provides solid evidence for the study of black holes [1,2,3,4,5]. Black hole spacetimes are very special, and the topological properties can be studied by the topological invariant Euler characteristic χ [6,7,8,9]. Black holes have some important features, which are easier to study by calculating the Euler characteristic. For example, black hole entropy has been discussed previously [9,10]; by using the Aharonov–Bohm effect, Padmanabhan emphasized the importance of the topological property of horizon temperatures [11]. Recently, Robson, Villari, and Biancalana [12] proposed a powerful method to study the topology of the Hawking temperature of black hole systems in two dimensions. The Hawking temperature of black holes in nontrivial metrics with different number of Killing horizons can be easily calculated by using topology method. This method has been effectively used to calculate the Hawking temperature of several four-dimensional black hole systems [13,14,15,16], which are well verified by the accuracy of the method.

In previous papers, the RVB method was only applied to spherical symmetrical black holes, for example, the Schwarzschild black hole, the Kerr black hole, the R-N metric in two dimensions [12], the Schwarzschild–de Sitter black hole [13], the anti-de Sitter black hole [14], the Schwarzschild-like black hole [15], and the BTZ black hole [16]. So, it would be interesting to consider the black hole which is not spherical symmetrical, and use the global monopole black hole as an example [17,18], which has deficit angle, and consequently, the spherical symmetry is broken. So, we consider whether the RVB method is applicable to the calculation of lower-dimensional (two-dimensional) global monopole black holes. It is also very useful for topologically understanding gravitational interaction in two-dimensional spacetime. After the calculation, we find that the Hawking temperature of the global monopole black hole in general relativity is easily obtained by the RVB method. The constant η also has an impact on the Hawking temperature, if the symmetry breaking scale η increases, the temperature decreases. We also discuss the Hawking temperature in massive gravity by the RVB method, and find that the temperature loses the information of the mass term; therefore, if we add an integral constant, which can be determined by the Hawking temperature obtained by the standard definition, then we will obtain the exact Hawking temperature for the massive global monopole black hole.

The structure of the paper is as follows. In Section 2, we introduce the RVB method, a topological formula for the Hawking temperature. In Section 3, we obtain the Hawking temperature of the global monopole black hole in general relativity by the RVB method. In Section 4, the Hawking temperature of the global monopole black hole in massive gravity is studied by the RVB method. Finally, Section 5 offers a brief conclusion of this paper.

## 2. RVB Method for the Hawking Temperature

Due to the diversity of black hole systems, stationary black holes and rotating black holes have simple metrics, so the temperature can be easily derived. However, for many special black holes, the temperature is difficult to calculate because of the complex coordinate system. Therefore, the RVB method relates the Hawking temperature of a black hole to the Euler characteristic χ; this is extremely useful in calculating the Hawking temperature in any coordinate system.

According to references [12,13], the Hawking temperature of a two-dimensional black hole can be found by the following topological formula:

(1) $$T_{H}=\frac{\hbar c}{4\pi \chi k_{B}}\sum_{j\leq \chi }\int_{r_{H_{j}}}\sqrt{|g|}Rdr,$$

where *ℏ* is the Planck constant, $k_{B}$ is the Boltzmann constant, *c* is the speed of light, *g* is the determinant of two-dimensional Euclidean metric, and $r_{H_{j}}$ is the location of *j*th Killing horizon. In this paper, we set that ℏ=1, c=1, and $k_{B}=1$. *R* is the Ricci scalar and depends on the spatial coordinate *r*; χ is the Euler characteristic, indicating the number of Killing horizons in the Euclidean geometry; and the symbol $\sum_{j\leq \chi }$ denotes a sum over all the Killing horizons.

The Euler characteristic χ is an important topological invariant, which describes information about the manifold structure and plays an important role as the link between geometry and topology. Here, the black hole spacetime with event horizon can be interpreted as a compact manifold. In *n* (even)-dimensions, the Euler characteristic χ of a geometry can be defined as [19]

(2) $$\chi =\frac{2}{\mathrm{area}\left(S^{n}\right)}\int_{M^{n}}\sqrt{|g|}d^{n}xG,$$

where $\mathrm{area}\left(S^{n}\right)$ is the surface area of a n-dimensional sphere, $M^{n}$ is a compact manifold. The density G in the Riemann coordinates can be defined as

(3) $$G=\frac{1}{2^{n/2}n!g}\varepsilon^{i_{1}\cdots i_{n}}\varepsilon^{j_{1}\cdots j_{n}}R_{i_{1}i_{2}j_{1}j_{2}}R_{i_{3}i_{4}j_{3}j_{4}}\ldots R_{i_{n-i}i_{n}j_{n-1}j_{n}}.$$

where $\varepsilon^{i_{1}\cdots i_{n}}$ is the Levi-Civita symbol, and $R_{\mu \nu \rho \tau }$ is the Riemann tensor. In two dimensions, the density becomes $G=\frac{R_{1212}}{g}=\frac{R}{2}$. In Ref. [12], the Euler characteristic is only calculated at the Killing horizon in the black hole.

In two dimensions for χ=1, the expression of the Euler characteristic becomes

(4) $$\frac{1}{4\pi T_{H}}\int_{r_{H}}\sqrt{|g|}Rdr=1,$$

which is expressed in (1) when the black hole has one Killing horizon.

## 3. RVB Method for the Hawking Temperature

Next, we will apply the topological formula, the RVB method, to study the Hawking temperature of the global monopole black hole. This is a non-spherical symmetrical black hole in four-dimensional spacetime, which has different topology from the spherical symmetrical spacetime, and the spherical symmetry is broken by a deficit angle. The global monopole black hole’s metric is [17,18]

(5) $$ds^{2}=-f\left(r\right)dt^{2}+h\left(r\right)dr^{2}+r^{2}d\theta^{2}+r^{2}\sin^{2}\theta d\varphi^{2},$$

where

(6) $$f\left(r\right)=h{\left(r\right)}^{-1}=1-8\pi G\eta^{2}-\frac{2MG}{r},$$

where *M* is the mass of the black hole, *G* is the Newtonian gravitational constant, and η is the symmetry-breaking scale. Because the metric (5) shows a space with a deficit angle, the area of a sphere of radius *r* is not $4\pi r^{2}$, but $4\pi \left(1-8\pi G\eta^{2}\right)r^{2}$ [18].

Due to definition, the Killing horizon does not involve the angular degree of freedoms, so we reduce the angular degree of freedoms of the spacetime. In Euclidean coordinate system, the two-dimensional line element of the global monopole black hole via the Wick rotation is t=iτ is [20]

(7) $$ds^{2}=f\left(r\right)d\tau^{2}+\frac{dr^{2}}{f(r)},$$

Therefore, the Ricci scalar from (7) is

(8) $$R=-\frac{d^{2}}{dr^{2}}f\left(r\right)=-\frac{4GM}{r^{3}}.$$

For a global monopole black hole, Equation (7) will diverge when f(r)=0. Then, considering f(r)=0, we derive the outer horizon $r_{H}=\frac{2MG}{1-8\pi G\eta^{2}}$ (the Killing horizon). If $\eta^{2}=\frac{1}{8\pi G}$, *r* is a singular point and has no physical meaning, then we find that r→∞ at the point with $\eta^{2}\to \frac{1}{8\pi G}$, which means that the whole space is within the event horizon of the black hole. The metric returns to the Schwarzschild black hole via $\eta^{2}=0$, so the symmetry breaking scale satisfy $0\leq \eta^{2}<\frac{1}{8\pi G}$. There is only one horizon, the Euler characteristic is also satisfies χ=1. Therefore, the global monopole black hole temperature is found by using Formula (1)

(9) $$T_{H}=\frac{1}{4\pi \chi }\int_{r_{H}}\sqrt{|g|}Rdr=\frac{{\left(1-8\pi G\eta^{2}\right)}^{2}}{8\pi MG}.$$

Using the RVB method, the Hawking temperature is inversely proportional to the mass of the global monopole black hole, while the constant η also has an impact on the Hawking temperature, if the constant η increases, then the temperature decreases.

## 4. Hawking Temperature of the Global Monopole Black Hole in Massive Gravity

In the calculation of the Hawking temperature, we also apply the RVB method in massive gravity. First, let us start with a brief introduction to the global monopole black hole in massive gravity. The three dimensional action of massive gravity with a *U(1)* gauge field is expressed by the following formula [21,22]:

(10) $$S=-\frac{1}{16\pi }\int d^{3}x\sqrt{-g}\left[R-2\Lambda +L\left(F\right)+{\tilde{M}}^{2}\sum_{i=1}^{4}c_{i}U_{i}\left(g,f\right)\right],$$

where *L(F)* is the Lagrangian function of the vector field, $\tilde{M}$ is the mass parameter, $c_{i}$ are some constants, and $\Lambda =-\frac{1}{l^{2}}$ is a negative cosmological constant. In this section, the effects of cosmic constants and Lagrangian are not considered, so we set *L(F)* and Λ both as 0. The symmetric polynomials of the eigenvalues computed by the d×d matrix $K_{v}^{u}=\sqrt{g^{u\alpha }f_{\alpha v}}$ is *U(i)*, which can be expressed as follows

(11) $$\begin{array}{c} U_{1}=\left[K\right],\\ U_{2}={\left[K\right]}^{2}-\left[K^{2}\right],\\ U_{3}={\left[K\right]}^{3}-3\left[K\right]\left[K^{2}\right]+2\left[K^{3}\right],\\ U_{4}={\left[K\right]}^{4}-6\left[K^{2}\right]{\left[K\right]}^{2}+8\left[K^{3}\right]\left[K\right]+3{\left[K^{2}\right]}^{2}-6\left[K^{4}\right]. \end{array}$$

where $\left[K\right]=K_{u}^{u}$ and ${\left(\sqrt{A}\right)}_{v}^{u}{\left(\sqrt{A}\right)}_{\lambda }^{v}=A_{\lambda }^{u}$. The metric follows the formula $f_{uv}=diag\left(0,0,a^{2}h_{ij}\right)$, with *a* is being a positive constant, $h_{ij}$ is the parameter for the space.

In massive gravity [22], there is an extra term in Formula (6),

(12) $$f\left(r\right)=1-8\pi G\eta^{2}-\frac{2MG}{r}+{\tilde{M}}^{2}cc_{1}r,$$

When f(r)=0, the global monopole black hole has two horizons

(13) $$r_{\pm }=\frac{\left(8\pi G\eta^{2}-1\right)\pm \sqrt{{\left(1-8\pi G\eta^{2}\right)}^{2}+8MG{\tilde{M}}^{2}cc_{1}}}{2{\tilde{M}}^{2}cc_{1}},$$

Only $r_{+}$ (the Killing horizon) is considered in this section.

The Ricci scalar of the global monopole black hole is

(14) $$R=-\frac{d^{2}}{dr^{2}}f\left(r\right)=-\frac{4GM}{r^{3}}.$$

This result shows that the Ricci scalar is equal to (8), which probably because the extra mass term in (12) does not have any contribution on the calculation of the Ricci scalar *R*. Then, by using the RVB method in (1), the calculated Hawking temperature of the global monopole black hole in massive gravity is expressed as

(15) $$T_{H}=\frac{GM}{2\pi r_{+}^{2}},$$

This is exactly the same as the temperature expression given in Section 3. However, by using the standard definition of [23], the Hawking temperature of the global monopole black hole in massive gravity is

(16) $${\tilde{T}}_{H}=\frac{f^{\prime }\left(r_{H}\right)}{4\pi }=\frac{GM}{2\pi r_{+}^{2}}+\frac{{\tilde{M}}^{2}cc_{1}}{4\pi }.$$

Comparing (15) and (16), we find that the Hawking temperature of the massive global monopole black hole calculated by the RVB method is different from that obtained by the standard definition. It seems to mean that the RVB method is not apply to the massive global monopole black hole. However, we should notice that the topological Formula (1) is actually an indefinite integral, because the indefinite integral has an integral constant, so the integral constant can be determined by the standard definition of the Hawking temperature, and we will obtain the exact Hawking temperature for the massive global monopole black hole. It means the influence of the mass term on temperature can not be ignored, and the temperature corrected by the standard definition [23] will be higher. Therefore, when the RVB method is used to calculate the Hawking temperature of a monopole black hole in massive gravity, it is necessary to correct the integral constant by the standard definition.

## 5. Summary and Conclusions

In this work, we studied the Hawking temperature of the monopole black hole, which is a non-spherical symmetrical black hole in four-dimensional spacetime, by using the Euler characteristic in topological formula Equation (1). The Hawking temperature is easily obtained by the RVB method, in the calculation of the Hawking temperature, Formula (9), and the integral of the temperature, Formula (9), where $r=r_{H}$ contains an integral constant, and the integral constant is 0. The temperature expression consistent with the method in quantum field theory of curved spacetime (the standard method mentioned in the paper). The result shows that the method is applicable to the global monopole black hole. However, when the RVB method is used to calculate the temperature in massive gravity, the result is not ideal. We find that we can obtain the same result as the standard method only when the integral constant is taken as a specific value [23].

## Data Availability

Not applicable.
